# Amino Acid Solutions for ^177^Lu-Oxodotreotide Premedication: A Tolerance Study

**DOI:** 10.3390/cancers14215212

**Published:** 2022-10-24

**Authors:** Pierre Courault, Agathe Deville, Vincent Habouzit, Frédéric Gervais, Claire Bolot, Claire Bournaud, Elise Levigoureux

**Affiliations:** 1 Hospices Civils de Lyon, Groupement Hospitalier Est, 69677 Bron, France; 2 Lyon Neuroscience Research Center, CNRS UMR5292, INSERM U1028, Université Claude Bernard Lyon 1, 69677 Bron, France; 3 Service de Pharmacie, Groupement Hospitalier Centre, Hospices Civils de Lyon, 69003 Lyon, France

**Keywords:** ^177^Lu-oxodotreotide, peptide receptor radionuclide therapy, premedication, amino acid perfusion

## Abstract

**Simple Summary:**

[^177^Lu]oxodotreotide (Lutathera^®^) was approved by the European Medical Agency in 2017 and the Food and Drug Administration in 2018 for the treatment of somatostatin receptor-positive gastroenteropancreatic neuroendocrine tumors as the first radiopharmaceutical for peptide receptor radionuclide therapy (PRRT). PRRT premedication using an amino acid solution is the standard regimen for renal protection. In this way, our nuclear medicine department used two types of amino acid perfusion. Firstly, a commercial solution containing a mixture of amino acids and, next, a lysine–arginine preparation. We aimed to estimate the tolerance profile and risk factors of adverse events with both solutions. In our large cohort of patients (76 patients for 236 cycles), we confirmed the better tolerance of the lysine–arginine preparation. The risk factors identified were being of the female sex and the use of the commercial solution. To our knowledge, this is the first study to evaluate the tolerance of amino acid solutions with real-life patient data in a large cohort.

**Abstract:**

Background: The co-infusion of amino acid solutions during peptide receptor radionuclide therapy reduces the tubular reabsorption of ^177^Lu-oxodotreotide, thus minimizing nephrotoxicity. In our nuclear medicine department, the patients received two different types of amino acid perfusion over time: a commercial solution (CS) containing 10% amino acids, and a 2.5% lysine–arginine (LysArg) hospital preparation, produced by a referral laboratory. The aim of the present study was to analyze the tolerance of the two amino acid solutions. Methods: The patient files were analyzed and double-checked. The study parameters comprised the gender, age, primary tumor site, type of amino acid perfusion, adverse events (AE) and WHO AE grades, antiemetic premedication, creatinine, and serum potassium level. Results: From February 2016 to February 2019, 76 patients were treated, for a total 235 cycles. AEs occurred in 71% of the CS cycles (*n* = 82/116), versus 18% (*n* = 21/119) in the LysArg group (*p* < 0.0001). In the CS group, the AEs were mostly WHO grade 4 (*n* = 24/82), and mostly grade 1 in the LysArg group (*n* = 13/21). Poisson regression showed a higher risk of AE overall and of grades 3 and 4 in the females and with CS. The mean creatinine clearance was identical before and after the PRRT cycles, whichever amino acid perfusion was used. Conclusions: The lysine–arginine preparation showed better tolerance than the commercial solution. The change to LysArg reduced the antiemetic premedication from four molecules to one.

## 1. Introduction

Neuroendocrine tumors (NETs) are part of a heterogeneous group of neoplasms developing from the neuroendocrine system. Their incidence and prevalence have been steadily rising in recent decades, due to better diagnostic methods, early-stage disease detection and improved survival [1]. According to a recent retrospective epidemiologic study by the National Cancer Institute’s Surveillance, Epidemiology and End Results (SEER) program, the incidence is around 6.98/100,000 per year [2]. The most common gastrointestinal NETs are those developed from the small intestine or rectum, pancreas and stomach or appendix. From 12% to 22% of patients are metastatic at presentation. Gastrointestinal NETs are associated with <50% 5-year survival in the cases of metastatic disease [3]. However, there are no prognostic classifications at the metastatic stage, except for the distribution of the metastatic extensions [4]. The survival for all NETs has improved over time, reflecting the improvement in therapies [5,6]. Recently, the experts suggested classifying NETs into two main categories, well or poorly differentiated, according to the mitotic and genetic features [7,8]. According to the current 2017 ENETS guidelines [9], the first-line systemic therapy usually consists of somatostatin analogues to control both the hormonal secretion and tumor growth. Other therapeutics have been developed: chemotherapy, targeted therapy or, in tumors overexpressing somatostatin receptors, peptide receptor radionuclide therapy (PRRT) [5,6,10,11]. The NETTER-1 study first showed that PRRT using ^177^Lu-oxodotreotide provided increased progression-free survival as compared to double-dose octreotide in patients with metastatic and progressive midgut NET [3]. Although the difference in the final overall survival was not significant, the 11.7-month difference seen in the median overall survival seems clinically relevant [12]. ^177^Lu-oxodotreotide is indicated for the treatment of unresectable or metastatic, progressive, well-differentiated (G1 and G2), somatostatin receptor-positive gastroenteropancreatic NET. However, the clinical studies also report adverse events (AEs) such as nephrotoxicity with PRRT. Thus, in the ^177^Lu-oxodotreotide summary of product characteristics, the administration procedure stipulates that an amino acid solution must be administered intravenously, 30 min prior to the ^177^Lu-oxodotreotide administration, for 4 h to prevent nephrotoxicity. The manufacturer recommends that the standard amino acid solution should be compounded with 25 g/L lysine and arginine in a 0.9% sodium chloride solution for injection.

Our nuclear medicine department used two types of amino acid perfusion. Firstly, due to the unavailability of a solution based only on lysine and arginine, and unduly restrictive manufacturing specifications by the French authorities, we performed the nephroprotection using a commercial solution (CS) containing a mixture of amino acids. However, due to the almost systematic digestive AEs, an alternative was subsequently developed. A lysine-arginine preparation (LysArg) was subcontracted to a laboratory and used in place of the CS. The present study describes the use of these two solutions in a large cohort of patients. The main aim of the study was to analyze the tolerance for the two amino acid perfusions.

## 2. Material and Methods

### 2.1. Patients

The patients were recruited from our nuclear medicine department. The treatment decision was approved by a multidisciplinary regional NET board (RENATEN), certified by the French National Institute of Cancer. The criteria for the PRRT comprised: a well-differentiated, progressive NET on the RECIST criteria, under octreotide treatment, in patients with a Karnofsky index ≥ 60, inoperable or metastatic tumors (grade 1 or 2, Ki67 < 20%), and an overexpression of somatostatin receptors. The exclusion criteria were similar to those in the NETTER-1 study, including a serum creatinine level above 150 μmol per liter, severe medullary insufficiency and hepatocellular failure, treatment with octreotide LAR, any surgery, liver-directed transarterial therapy, or chemotherapy within 12 weeks before randomization [3]. The PRRT consisted of four cycles of ^177^Lu-oxodotreotide every 8 weeks. After providing informed consent, the patients treated by ^177^Lu-oxodotreotide PRRT for somatostatin receptor-positive gastroenteropancreatic NET between February 2016 and February 2019 were included. In accordance with French legislation, all the patients were informed about the use of their clinical data. One patient declined the collection of his data.

### 2.2. Data Collection

The clinical and paraclinical data were recorded prospectively during the hospital stay, then analyzed retrospectively with double-checking by two operators. The pre-treatment data were recorded the day before the PRRT. The clinical data were collected from the paper or computerized medical files and nursing traceability forms. The patients were monitored throughout their hospital stay, and all the AEs were recorded. The paraclinical data comprised: a pre-treatment electrocardiogram (EKG) and blood sample (complete blood count, serum electrolytes, creatinine and creatinine clearance and liver function). A post-treatment EKG was performed the day after the PRRT. The same biological parameters were assessed twice a month after discharge. The study parameters comprised: gender, age, primary tumor site, type of amino acid perfusion, liver metastasis, AEs and WHO grades, antiemetic premedication, creatinine clearance and kalemia.

### 2.3. Assessment of Adverse Events

In the light of the ERASMUS and NETTER-1 studies [3,12,13], the following AEs were systematically investigated: nausea/vomiting, diarrhea, flush, headache and nephrotoxicity. The most frequent were graded on the WHO scale [14]: nausea/vomiting (grade 0 = none; 1 = nausea; 2 = transient vomiting; 3 = treatment-seeking vomiting; 4 = intractable vomiting), diarrhea (grade 0 = none; 1 = transient < 2 days; 2 = tolerable > 2 days; 3 = intolerable requiring treatment; 4 = dehydration/hemorrhagic diarrhea), flushes (grade 0 = none; 1 = mild; 2 = moderate; 3 = interfering with activities of daily life) and headache (grade 0 = none; 1 = mild; 2 = moderate, interfering with usual functioning; 3 = impaired activities of daily living). The AE grade data were analyzed and checked double-blind; in case of a discrepancy, a third evaluation was performed.

### 2.4. Amino acid Composition

**Figure 1:**
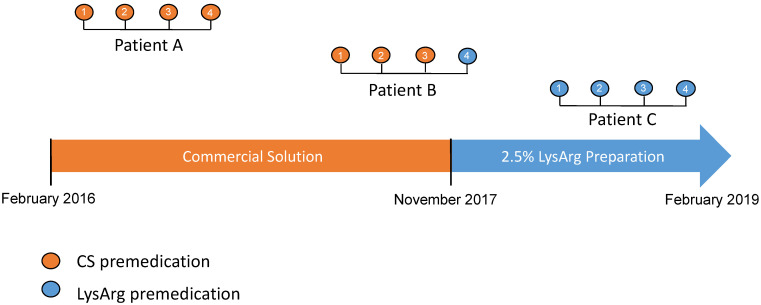
Flow-chart representing timeline use of different amino acid solutions (CS: commercial solution; LysArg: L-lysine L-arginine). From February 2016 to February 2019, all patients were treated with CS solution. After November 2017, all patients were treated with LysArg preparation. PPRT consisted of 4 cycles (1 to 4) of [^117^Lu]oxodotreotide every 8 weeks.

Two types of amino acid solutions were administered during the study period. The first, used from February 2016 to November 2017, was a commercial solution only (CS) containing a 10% amino acid mixture. After November 2017, the second was a 2.5% L-lysine and L-arginine hospital preparation only (LysArg), produced by a referral laboratory. Figure 1 summarizes the timeline use of the amino acid solutions. The amino acid solutions were administered according to the recommendations, i.e., 30 min prior to the ^177^Lu-oxodotreotide administration, for 4 h to prevent nephrotoxicity. The details of the composition and characteristics of these solutions are summarized in Table 1.

### 2.5. Data Analysis

Student’s *t*-tests assessed the intergroup homogeneity for age and antiemetic use. Chi-square tests compared the primary tumor site distribution and AE WHO grade distribution. Chi-square tests with Yates’ correction compared the gender. Fisher exact tests compared the AE grade frequency. A Poisson regression model assessed the influence of various factors (gender, age, amino acid solution, number of cycles, presence of liver metastasis) on the occurrence of AE (any grade) versus no AE, and serious AE (grades 3 and 4) versus other conditions (no AE, or grade 1 or 2), overall and on the subgroup analysis, according to the amino acid solution (CS, LysArg). The results were expressed as the relative risk (RR). Chi-square tests with Yates’ correction assessed the intergroup differences in the onset of transient kidney failure or dyskalemia. For all the analyses, the significance threshold was set at *p* < 0.05.

## 3. Results

From February 2016 to February 2019, 76 patients (M/F sex ratio = 1.22) were treated, for a total 235 cycles. Two groups were distinguished, according to the nephroprotective premedication: 46 patients received ≥1 LysArg administration, for a total of 119 cycles, and 40 received ≥1 CS administration, for a total of 116 cycles. Ten patients received both solutions. No statistical differences were found between the groups for age (*p* = 0.678), gender (*p* = 0.531) and primary site (*p* = 0.884). Table 2 and Table 3 show the epidemiologic data and primary site distribution, respectively, per group.

### 3.1. Adverse Events

**Figure 2:**
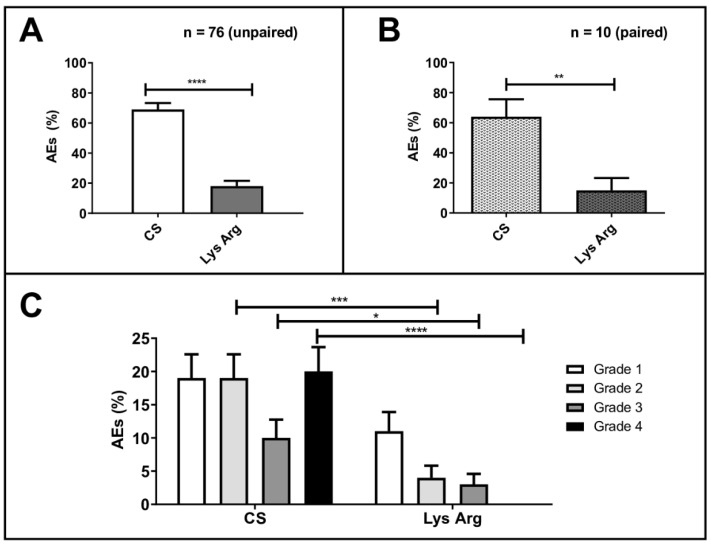
Percentage adverse events (AEs) in all patients (*n* = 76) treated with the commercial solution (CS) or the lysine–arginine preparation (LysArg) (**A**), in 10 patients who received both solutions (**B**) and distribution according to WHO grade (**C**). Significant levels: * *p* < 0.05; ** *p* < 0.01; *** *p* < 0.001 and **** *p* < 0.0001. Bars plot SD values.

AEs occurred in 71% of the cycles (*n* = 82/116) in the CS group, versus 18% (*n* = 21/119) in the LysArg group (*p* < 0.0001; Figure 2A,B). The most frequent were nausea and vomiting (*n* = 83/103), flush (*n* = 9/103), diarrhea (*n* = 6/103) and headache (*n* = 5/103). One patient declined to continue the PRRT after the first cycle, due to grade 4 vomiting after the CS injection. Table 4 summarizes the individual adverse events that occurred in the patients, per group, and the statistical subgroup analyses, per the adverse event.

**Figure 3:**
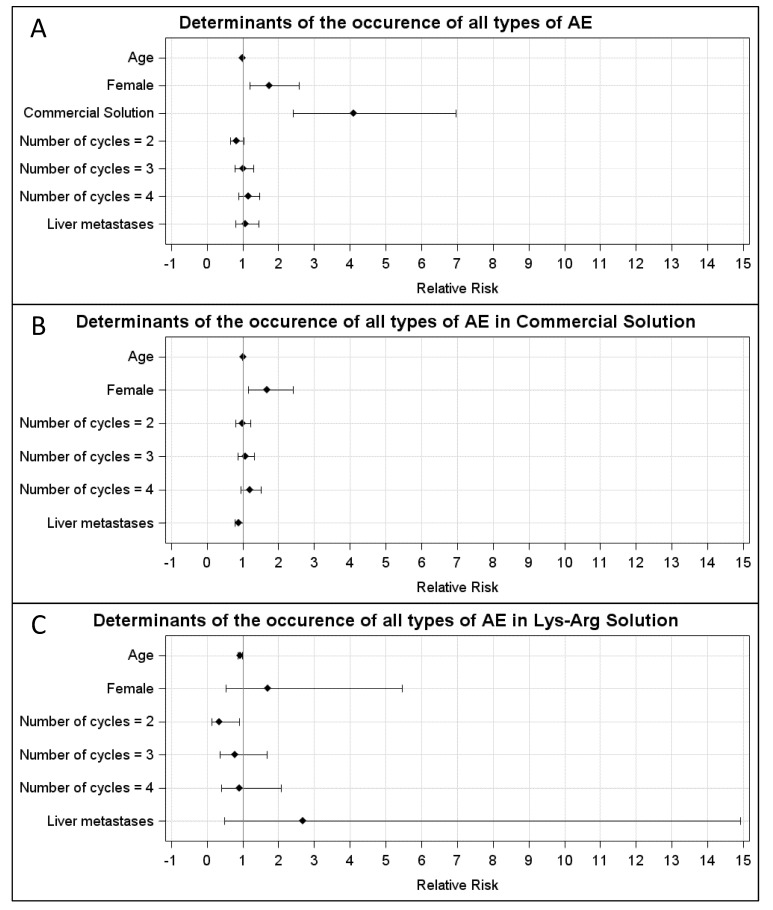
Relative risk calculated with Poisson regression for (**A**) all types of AE in the LysArg group, compared to the CS group; (**B**) all types of AE in the CS only and (**C**) LysArg only. Determinants were age, gender, number of cycles and presence of liver metastasis.

**Figure 4:**
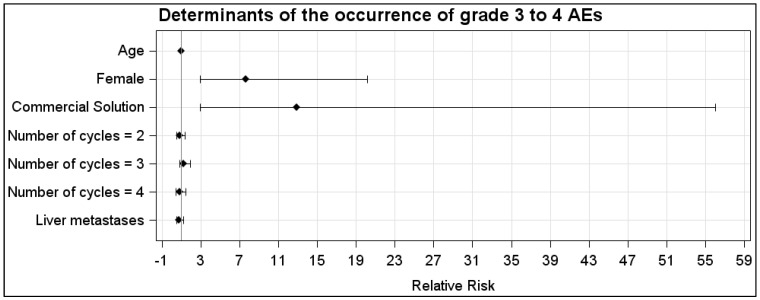
Relative risk calculated with Poisson regression for grades 3 and 4 in the LysArg group compared to the CS group. Determinants were age, gender, number of cycles and presence of liver metastasis.

In the CS group, the AEs were WHO grade 1, 2, 3 and 4 in, respectively, 20% (23/116), 20% (23/116), 10% (12/116) and 21% (24/116) of the cycles. In the LysArg group, the AEs were grade 1, 2 and 3 in, respectively, 11% (*n* = 13/119), 4% (*n* = 5/119) and 3% (*n* = 3/119) of the cycles. Figure 2C displays the significant intergroup difference in the AE distribution (*p* = 0.008). Significant differences in frequency were found for AE grades 2, 3 and 4 (*p* = 0.0002, 0.016 and <0.0001, respectively) but not for grade 1 (*p* = 0.07). Interestingly, 10 patients, for a total of 36 cycles, received both solutions; 65% (*n* = 11/17) of the CS cycles were complicated by an AE, versus 16% of the LysArg cycles (*n* = 3/19) (*p* = 0.0077) (Figure 2B). Poisson regression showed a higher risk of AE of any grade in the females (RR = 1.75; 95%CI, 1.19–2.57) and with CS (RR = 4.09; 95%CI, 2.41–6.95) (Figure 3A). In the CS sub-group analysis (Figure 3B), Poisson regression again showed higher risk of an AE in the females (RR = 1.67; 95%CI, 1.15–2.41). In the LysArg subgroup analysis (Figure 3C), the gender showed no influence on the AEs, and the patients experienced significantly fewer AEs in the second than in the first cycle, showing the second cycle to be a protective factor (RR = 0.35; 95%IC, 0.13–0.91). Poisson regression showed a higher risk of a grade 3 or 4 AE in the females (RR = 7.64; 95%CI, 2.90–20.18) and with the CS (RR = 12.90; 95%CI, 2.97–56.00) (Figure 4). No differences emerged according to the number of cycles, presence of liver metastasis or age.

### 3.2. Paraclinical Parameters

Short-term follow-up of nephrotoxicity showed that three patients (one in the CS group and two in the LysArg group) experienced a transient aggravation of kidney failure, from stage 2 to stage 3. A post-PRRT dyskalemia was observed in 31 and 40 cycles for the LysArg and CS, respectively (*p* > 0.05), mainly in the form of hyperkalemia (26 out of 31, and 36 out of 40 cycles). No difference was found between the pre- and post-PRRT EKGs.

### 3.3. Antiemetic Use

The antiemetic premedication combined ondansetron, methylprednisolone, clorazepate, aprepitant, alprazolam, hydroxyzine and metoclopramide. In the CS group, the mean antiemetic use was 3.3 molecules, versus 1.4 in the LysArg group (*p* < 0.0001).

## 4. Discussion

The present study compared the tolerance for two amino acid solutions to prevent the nephrotoxicity induced by ^177^Lu-oxodotreotide PRRT. Short-term nephroprotection was equivalent for both solutions. However, the LysArg preparation dramatically reduced the onset of clinical AEs as compared to the CS. This may be explained by various factors discussed below: predisposing factors according to group, and the amino acid solution composition and osmolarity.

As described previously, nausea and vomiting were the most common clinical AEs. In a review, Warr reported the well-established risk factors for chemotherapy-induced emesis [15]: vomiting during the previous cycle, the type of chemotherapy, type of antiemetic, gender, age, alcohol consumption and pregnancy. In the present study, only vomiting during the previous cycle, the gender, presence of liver metastasis and age were relevant; the first-line antiemetics were similar in all the patients and pregnancy was a contraindication to the PRRT. Poisson regression showed that the gender and type of amino acid were the only two factors influencing an AE onset. Roila showed that emesis in chemotherapy becomes more likely with successive cycles [16], but the present study did not find similar results, excluding a cycle-number effect. Interestingly, the LysArg subgroup analysis showed that cycle 2 had a protective effect on an AE onset compared to cycle 1. This result should be interpreted with caution, due to the possible confounding factor of the adaptation of antiemetic treatment after cycle 1, rather than a real cycle effect. The gender effect was also not consistent in the subgroup analysis: for CS, being female was a risk factor for AEs, whereas the LysArg was not influenced by gender. The group demographics showed no differences, excluding any selection bias. Thus, the LysArg preparation should be preferred for all patients, and especially for females.

Concerning the composition of the amino acid solutions, the CS was our first choice in the absence of other approved formulae. Its main indication is for parenteral nutrition, and CS thus contains amino acids that are wholly non-contributive for nephroprotection in PRRT. Some are known to have an influence on the lower esophageal sphincter and induce nausea and vomiting [17]. For example, aromatic amino acids, such as tryptophan and phenylalanine, have a direct effect on the gastric wall [18]. In addition, unnecessary amino acids increase the osmolarity of the solution. It is well known that hyperosmolar solutions lead to many adverse effects, such as nausea and vomiting [19,20] or hyperkalemia [21,22]. Finally, the CS was administered at a flow rate of 250–500 mL/h, much higher than that recommended for parenteral nutrition, for which it should be administered over a minimum of 12 h. In contrast, the LysArg was developed expressly for ^177^Lu-oxodotreotide premedication and contains only amino acids useful for nephroprotection: L-lysine and L-arginine. Radiolabeled somatostatin analogues undergo reuptake in the proximal tubules of the kidney [23]. A co-infusion with amino acid solution during PRRT aims to decrease this reuptake, for which L-arginine and L-lysine have been demonstrated to be effective [19,24,25], as they undergo glomerular filtration and, via competition, interfere with the renal resorption of the somatostatin analogues [26]. The mechanism involves competition between the positive charges of the amino acid or radiolabeled octreotide, binding the negative charge of the renal tubule cells [27,28,29]. Several clinical and preclinical studies have suggested that megalin protein may be involved [30,31,32]; this negative protein is known to bind with and take up cationic compounds [33]. Therefore, a co-infusion with L-arginine and L-lysine significantly reduces the kidney exposure and risk of kidney failure during PRRT. The use of specific amino acids reduces osmolarity, which, in our opinion, underlies the lower AE rate in the present study, with a positive impact on the quality of life (QoL). In the modern integrative approach, health-related QoL counts as a favorable outcome, or even a crucial endpoint, in evaluating new cancer treatments. Reducing nausea and vomiting significantly improves wellbeing, with clinical benefits [34,35].

Interestingly, in the NETTER-1 study, nausea and vomiting occurred in 59% (*n* = 65/111) and 47% (*n* = 52/111) of the cycles. However, most of these cases were attributed to the particular amino acid solutions used [3]. The solutions in the NETTER-1 study had characteristics similar to the CS used in the present study (i.e., unnecessary for nephroprotective purpose, with high osmolarity). Our results showed similar AE rates using the CS to those observed in NETTER-1. Therefore, the AEs may be largely due to the amino acid composition. Double-checking data by two operators showed a match rate of 73.5%, confirming accurate AE collection and evaluation; discrepant data were discussed to reach the correct classification.

Concerning serum potassium, the dyskalemia mainly consisted of hyperkalemia and did not differ between groups. The presence of lysine in both solutions could explain this hyperkalemia. As described in other studies consistent with the present results, lysine can induce hyperkalemia by ketogenic acidosis [19,20,22]. However, the dyskalemia was not associated with clinical or EKG signs. Other causes than amino acids could be considered for hyperkalemia, such as tumor cell death.

The LysArg preparation significantly reduced the antiemetic use, from four molecules to only one (ondansetron). Because of their various pharmacodynamic actions, antiemetics are implicated in many unsafe drug–drug interactions [36]. Economically, the reduction was not significant, due to the relatively low cost of these drugs. However, the hospital stay was shortened by reducing the AE rate, and thus the necessity of follow-up. It should be borne in mind that the LysArg preparation is more expensive than the CS. Limitations can be raised in this study. The influence of clinical data such as liver tumor burden or performance status were not evaluated, despite the fact that they might influence the results. The PS was not recorded and evaluated according to the subgroups; all the patients treated had a Karnofsky index ≥ 60%, as required according to the NETTER inclusion criteria. The liver tumor volume could not be addressed in this cohort, constituted of patients referred from various centers: MRI or CT imaging was not always available for a centralized assessment. Furthermore, it was not required in daily practice, as far as it has been demonstrated that it does not influence PRRT efficacy [37]. It may nevertheless have an influence on the AA solution tolerance.

The present study demonstrated the superiority of the LysArg preparation in terms of an AE onset. However, the prime aim of amino acid perfusion is to reduce the long-term risk of kidney failure. We previously reported that L-lysine and L-arginine were the only amino acids needed for nephroprotection, but this study did not conclude whether the LysArg or CS protects the patient against long-term PRRT nephrotoxicity. Further data and studies are required to compare the long-term nephroprotection between these two solutions. The present results highlight the need to use an adequate amino acid perfusion as a ^177^Lu-oxodotreotide premedication. Although the AE rate was lower with the LysArg, it was still 18%. Regarding the NETTER-1 study, and excluding the AEs caused by the amino acid perfusion, the rates of AEs caused by the PRRT were 20% for nausea (*n* = 23/111) and 13% for vomiting (*n* = 14/111). Interestingly, a recent study using LysArg as a premedication also showed important differences in vomiting compared to the NETTER-1 study (8% vs. 47%) [38]. Thus, the 18% AE rate observed with the LysArg preparation in the present study could be mainly attributed to the ^177^Lu-oxodotreotide administration, rather than to the amino acid solution.

## 5. Conclusions

The LysArg preparation significantly reduced the occurrence of AEs; the AEs were also less severe, mostly grade 1 or 2 on the WHO scale. The change in amino acid premedication also reduced the antiemetic use. The LysArg preparation has been authorized by the European Medicines Agency for use in the European Union.

## Figures and Tables

**Table 1:** Composition of the two solutions.

Characteristics		Commercial Solution 10%	Lysine-Arginine 2.5% Preparation
**Amino Acid Composition (g/L)**	L-Lysine	11.00	25
	L-Arginine	8.40	25
	L-Isoleucine	6.70	-
	L-leucine	10.00	-
	L-Valine	7.60	-
	L-Methionine	2.40	-
	L-phenylalanine	4.20	-
	L-Threonine	3.70	-
	L-Tryptophan	2.00	-
	L-Histidine	3.80	-
	L-Alanine	8.00	-
	L-Aspartic acid	6.00	-
	L-Cysteine	1.89	-
	L-Glutamic Acid	10.00	-
	Glycine	4.00	-
	L-Proline	3.00	-
	L-Serine	4.00	-
	L-Tyrosine	0.45	-
	L-Ornithine hydrochloride	3.18	-
	L-Taurine	0.60	-
**Osmolarity (mOsm/L)**		780	420–480
**Volume administered (L)**		2	1

**Table 2:** Demographic data. There were no significant differences between groups for age and sex-ratio.

Amino Acid Group	LysArg Preparation	Commercial Solution	Total
*n* patients	46	40	76
*n* cycles	119	116	235
Mean Age	64 ± 9	65 ± 11	64 ± 10
Gender M/F	27/19	20/20	42/34

**Table 3:** Primary tumor site distribution in the two groups (*n* = 86; 10 patients received both solutions and are counted in both groups).

Primary Tumor Site	LysArg Preparation	Commercial Solution	Total
Intestine	32 (70%)	30 (74%)	62 (72%)
Pancreas	7 (15%)	6 (15%)	13 (15%)
Bronchi	3 (6.5%)	2 (5%)	5 (6%)
Rectum	3 (6.5%)	1 (2.5%)	4 (5%)
Unknown	1 (2%)	1 (2.5%)	2 (2%)

**Table 4:** Individual adverse event occurring in patients per group.

Adverse Events	LysArg Preparation	Commercial Solution	*p*-Value	Total
Nausea-vomiting	13 (11%)	70 (60.5%)	<0.001	83
Flush	6 (5%)	3 (2.6%)	0.50	9
Diarrhea	2 (2%)	4 (3.4%)	0.44	6
Headache	0	5 (4.5%)	0.02	5
Total	21 (18%)	82 (71%)	<0.001	103

## Data Availability

The data presented in this study are available on request from the corresponding author.
